# Systematic review on serotypes distribution of pneumococcal pneumonia in adults and the elderly

**DOI:** 10.1186/s12889-025-22164-x

**Published:** 2025-03-29

**Authors:** Fatiha Mrabt, Sandra Guedes

**Affiliations:** Sanofi Pasteur, Lyon, France

**Keywords:** *Streptococcus pneumoniae*, Pneumococcal pneumonia, Serotype, Pneumococcal vaccines, Adults

## Abstract

**Background:**

Pneumococcal pneumonia is a major cause of morbidity and mortality among adults, especially those over 65 years of age. Understanding pneumococcal serotype-specific epidemiology in adults and elderly is necessary to inform vaccination policies and to guide the inclusion of serotypes in pneumococcal vaccines. This study aimed to identify the serotypes causing pneumonia in the elderly.

**Methods:**

A systematic review search was performed using the PubMed database from 1984 to 2020. The search was limited to articles written in English. Studies assessing pneumococcal pneumonia in adults were included. Meta-analysis, other systematic literature reviews and case-reports were excluded. Studies published after the introduction of vaccines (PPSV23 and PCVs) were included.

**Results:**

Forty studies were included. The most common serotype identified in the majority of the articles in adults was the serotype 3 followed by serotypes 19A and 11A. Those serotypes are included in current vaccines. Emergence of non-vaccine serotypes was also observed.

**Conclusion:**

Pneumococcal pneumonia remains a high burden in the elderly despite the existence of vaccines for many years. In 2019, nearly 1.4 million deaths were attributable to pneumococcal pneumonia (50% of whom were over 70 years old) and was the leading cause of deaths from infectious disease worldwide. The study highlights the importance of constant monitoring serotypes emerging in this population to better target vaccination strategies.

**Supplementary Information:**

The online version contains supplementary material available at 10.1186/s12889-025-22164-x.

## Introduction

Community-acquired pneumonia (CAP), defined as pneumonia acquired outside the hospital, is globally one of the leading causes of lower respiratory infection (LRI), morbidity and mortality in the very young and in the elderly [1]. LRI are a major public health problem worldwide, and was the fourth leading cause of death in 2019, with 2.6 million of deaths [2]. *Streptococcus pneumoniae* (*S. pneumoniae*) has been recognized as the most common pathogen of CAP, causing more deaths than all other etiologies combined [3, 4].

*S. pneumoniae* is a Gram-positive coccus bacterium with more than 90 serotypes already identified. Only a few serotypes are, however, responsible for most of the cases causing pneumococcal infections; it has been estimated that about 62% of invasive pneumococcal disease (IPD) was caused by 10 serotypes worldwide [5].

The prevalence and distribution of *S*. *pneumoniae* serotypes may differ across populations, geographical areas and over time.

The majority of pneumococcal pneumonia deaths can be prevented through vaccination [6].

Currently, there are two different types of pneumococcal vaccines licensed for the elderly: a pneumococcal polysaccharide-based vaccine (PPSV23) and pneumococcal conjugate vaccines (PCVs). National vaccine recommendations may differ per region and country.

The first pneumococcal vaccine, PPSV23, was licensed in 1983, and contains polysaccharide antigen of 23 types of pneumococcal bacteria that cause 60–76% of invasive disease [5].

It contains the following capsular serotypes: 1, 2, 3, 4, 5, 6B, 7F, 8, 9N, 9 V, 10A, 11A, 12F, 14, 15B, 17F, 18C, 19F, 19A, 20, 22F, 23F, and 33F.

The PPSV23 is poorly immunogenic in infants under 2 years of age, and the immunogenicity seems to decline with age; the immune system undergoes characteristic changes, which lead to functional deficits and dysregulation of most immune mechanisms, so the effectiveness of this vaccine is lower in the elderly and the youngest because it cannot induce an adequate immune response. The poor immunogenicity of PPSV23 led to the development of pneumococcal conjugate vaccines (PCVs), which can induce a stronger immune response than polysaccharide vaccine due to their ability to elicit memory T cell response.

The first PCV licensed was a 7-valent pneumococcal vaccine (PCV7), and it was introduced in the United States for the routine immunization of children in 2000. PCV7 includes purified capsular polysaccharide of seven serotypes of *S. pneumoniae* (4, 9 V, 14, 19F, 23F, 18C, and 6B) conjugated to a nontoxic variant of diphtheria toxin known as CRM_197._

After its introduction in the US, IPD decreased by 76% in children under the age of five, and also among unvaccinated children and adults due to indirect vaccine protection [7]. However, the incidence of IPD due to non-vaccine serotypes (NVT) increased by 22%, particularly serotype 19A [8–10].

PCV7 was replaced by the 10-valent (PCV10) and 13-valent pneumococcal conjugate vaccines (PCV13) thereafter [11].

At the time of this study, there were two PCVs in the global market—PCV10 and PCV13.

PCV10 has been licensed in 2009 and it includes the same serotypes as PCV7 plus the additional serotypes 1, 5 and 7F.

PCV13 licensed in 2010, and it includes the 7 serotypes of PCV7 plus serotypes 1, 3, 5, 6A, 7F and 19A which are also conjugated to CRM_197_.

It was estimated that approximately 20%–25% of IPD cases and 10% of CAP cases in adults aged ≥ 65 years are caused by serotypes included in PCV13 and could be potentially preventable with the use of PCV13 in this population [5].

In 2014, the US Advisory Committee on Immunization Practices (ACIP) recommended the use of PCV13 with PPSV23 for adults aged > 65 years [12, 13].

In 2021, two new vaccines were launched on the global market: PCV15 and PCV20.

Since October 2021, the ACIP recommended now the use of PCV15 followed by PPSV23 or PCV20 alone for PCV-naïve adults aged ≥ 65 years for the prevention of IPD and pneumonia [14]. In Europe, vaccination recommendations differ from one country to another [15].

Many countries have implemented pneumococcal vaccination programs for infants and older adults. Whereas vaccination programs for adults (with PPSV23) have resulted in relatively low vaccination rates, introduction of childhood PCV immunization has been associated with a reduction of both IPD and carriage vaccine serotypes.

Following the introduction of PCVs, a decrease in overall and vaccine-serotypes IPD has also been reported among adults and unvaccinated children, suggesting an indirect protection, from the PCV vaccination in children.

This indirect (herd) protection results from a reduced vaccine-serotype carriage among vaccinated individuals who then have a lowered chance of transferring these serotypes to non-vaccinated individuals [16].

Parallel to the reduction in serotypes included in PCVs, an increase in IPD caused by non-PCV serotypes – known as serotype replacement, was observed in many countries [17, 18]. This suggests that, by reducing the prevalence of vaccine serotypes, a vacant niche has been created which may be filled by non-vaccine serotypes. Thus, the vaccine’s effectiveness may decrease over time, if the serotype formulation of PCV is not continually reevaluated [19].

Most data on the sero-epidemiology have been collected from invasive isolates such as IPD. Pneumococcal pneumonia is a significant cause of morbidity and mortality among the elderly, and it is important to understand which serotypes are causing most of the disease in this population to better support new vaccines development and recommendations of use.

The aim of this review was to determine which serotypes were the main serotypes causing pneumococcal pneumonia in the elderly and to assess if they were covered by approved pneumococcal vaccines and thus guide vaccination recommendations. Both data on bacteremic and non-bacteremic pneumococcal pneumonia were included.

The aim of this study was to estimate the burden of pneumococcal pneumonia and the effect of current vaccination programs on the sero-epidemiology of the disease in the elderly, with a focus on serotypes not covered by existing vaccines (PPSV23 or PCVs). In the absence of elderly-specific information, the study provides information on adults.

Specific objectives were:

- To identify the main serotypes responsible for pneumococcal pneumonia in elderly
- To summarize available data on the proportion of non-vaccine serotypes of pneumococcal pneumonia

## Methods

This literature review aimed to systematize relevant studies on the burden of pneumococcal pneumonia caused by non-pneumococcal vaccine serotypes in adults and the elderly. We conducted this systematic review in accordance with the Preferred Reporting Items for Systematic Reviews and Meta-Analyses (PRISMA) reporting guidelines [20] (PRISMA checklist, Supplementary Table S7).

### Criteria for selecting studies for this review

#### Types of studies included

Peer-reviewed studies reporting data on serotypes causing pneumococcal pneumonia in adults^1^ and in the elderly (adults from 65 years of age).

Meta-analysis, other systematic literature reviews and case-reports were excluded from this review but information from individual studies were included if available and relevant.

The PICO framework elements presented in Table 1 above, were used to identify and select studies to be included in this review.

**Table 1:** PICO framework for study inclusion

What is the burden of pneumococcal pneumonia due to serotypes not included in a pneumococcal vaccine in the elderly?	
P	Adults of either sex, aged 65 years and above, living worldwide, and having been diagnosed with pneumococcal pneumonia
I	Not applicable (burden of disease). Population can be vaccinated or not vaccinated
C	Not applicable (burden of disease)
O	Studies reporting the following outcomes: Proportion and distribution of serotypes responsible for pneumococcal pneumonia, stratified by vaccine serotype (PCV/PPSV23 STs) or non-vaccine serotype (NVTs: non-PCV/non-PPSV23 STs)

#### Exclusion criteria

Studies with any of the following criteria were excluded from the review:

- studies reporting only pediatric population
- studies focusing only on non-pneumococcal pneumonia
- studies reporting exclusively on invasive disease (except for invasive pneumococcal pneumonia) (more generally the studies with non-pneumonia outcomes)
- studies published before the introduction of a pneumococcal vaccine (PPSV23 or PCVs)
- experimental studies (e.g., animal models)
- commentary articles, case-reports, conference abstracts

### Search methods for identification of studies

The literature search was performed using the PubMed database. Only studies in English language were included in this review. A combination of free-text and controlled vocabulary terms related to the burden of pneumococcal pneumonia and serotypes implicated was used to develop a sensitive search strategy. Studies published after the introduction of vaccines (PPSV23 and PCVs) were included (studies from 1984 to 2020). Children were excluded from the search.

Search detail is provided in Supplementary Table S1.

#### Study selection

Studies were selected in two steps. Titles and abstracts were initially screened to identify relevant references from search strategy by both the authors independently. If title and/or abstract provided insufficient information to assess the relevance or if a final decision could not be made, the full article was reviewed. Then, full texts of articles selected in the first stage were independently reviewed for final inclusion. Any discrepancies in study selection were resolved by discussion. Duplicates and irrelevant studies were excluded.

### Data extraction

Data were extracted into a spreadsheet. Relevant data from the final studies screened were inserted into a summary table; the following information was extracted from each included study:

- Study identification: name of the first author, year of publication, country.
- Study characteristics: study design (e.g., cohort, clinical trial), setting (hospital, outpatient), study period, sample size, age of participants.
- Outcome measures of interest: distribution and proportion of vaccine and non-vaccine serotypes responsible for pneumococcal pneumonia in the elderly

### Data analysis

In this review, the primary outcome was pneumococcal pneumonia caused by non-vaccine serotypes in adults with a focus in the elderly. In countries with pediatric and adult vaccination programs, the direct vs indirect impact of vaccines is hard to differentiate so the results are presented together. A narrative approach was used to synthesize the findings.

## Results

### Study selection

After exclusion of duplicates, a total of 305 potentially relevant articles were included. After screening abstracts and titles, 221 articles were excluded, leaving 84 articles for full text review. Forty-four articles were further excluded for the following reasons: wrong outcomes (25 articles), wrong patient population (9 articles), wrong study design (5 articles) and wrong disease entity (5 articles) (Supplementary Table S2).

Finally, 40 articles were eligible for inclusion in this review [21–60].

**Fig. 1:**
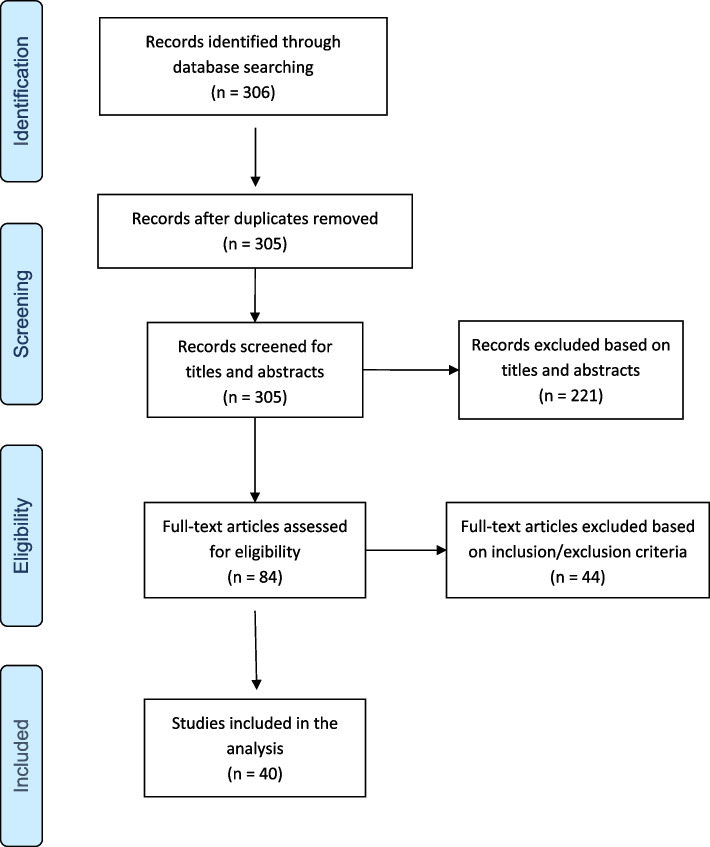
Flow chart for the systematic search

The PRISMA flow diagram (Fig. 1) provides detailed information regarding the selection process of studies.

### Study characteristics

A total of 40 studies were identified, covering 18 countries, mostly in Europe (67%). All studies were from hospitalized patients. Main characteristics of studies included are summarized in Supplementary Table S3.

Studies were heterogeneous in terms of design, methods, interventions, and outcome measurements.

Most data were from Spain (10 studies) followed by the UK (5 studies), Japan (4 studies), Sweden (3 studies), US, Canada, Denmark, and Netherlands (2 studies each). Data from single studies in Australia, Chile, France, Greece, India, Mexico, Poland, Portugal, Scotland and South Korea, were also assessed.

Most studies (71%) were cohort studies (22 prospective studies, 6 retrospective studies and 1 study analyzed data from 2 cohorts (a prospective cohort 1987–1998 and a retrospective cohort 1999–2008).

Included studies were published between 1984 and 2020 (with data collection between 1977 and 2018). Most studies included different age groups, and only 2 studies were conducted exclusively in elderly subjects.

Twenty-five studies included data from invasive pneumococcal pneumonia (IPP) and non-invasive pneumococcal pneumonia (NIPP), three of which, distinguished between invasive and non-invasive pneumococcal serotypes. Six studies included data only from NIPP and nine from IPP (Supplementary Table S4).

Thirteen publications reported on the vaccination status and thirty-four on the serotyping method (Supplementary Table S3).

### Synthesis of results

**Fig. 2:**
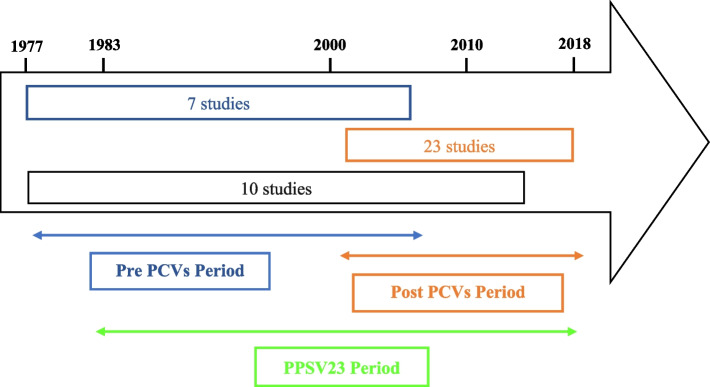
Distribution of the included studies according to the study period when the were conducted

Distribution of studies by PCV vaccination period is shown in Fig. 2. Most studies (*N* = 23 studies, 57%) were conducted in the post-PCVs period (2001–2018). Ten studies were conducted in an overlapping period (including pre- and post-PCV periods) and seven studies were conducted in the pre-PCVs period.

Periods were defined from the introduction of PCVs into childhood immunization: seven studies were conducted before the introduction of PCV7 (Pre-PCV period) [28, 29, 32, 36, 38, 39, 59] and twenty-three studies in the post-vaccination period (Post-PCVs period) [21–23, 25–27, 30, 33, 35, 40–43, 46, 48–52, 55–57, 60].

Vaccination policies differ by country, so the introduction of PCVs was not done at the same time. The dates of introduction of PCVs into childhood immunization in the countries included in our study are summarized in the Supplementary Table S5.

### Distribution of pneumococcal pneumonia serotypes

Almost 30,000 pneumococcal caused CAP isolates were available for analysis (*N* = 28,439), and most of these CAP isolates had information on serotypes (N_ST_ = 23,235).

The most prevalent pneumococcal serotypes for each study are presented in Supplementary Table S3.

**Fig. 3:**
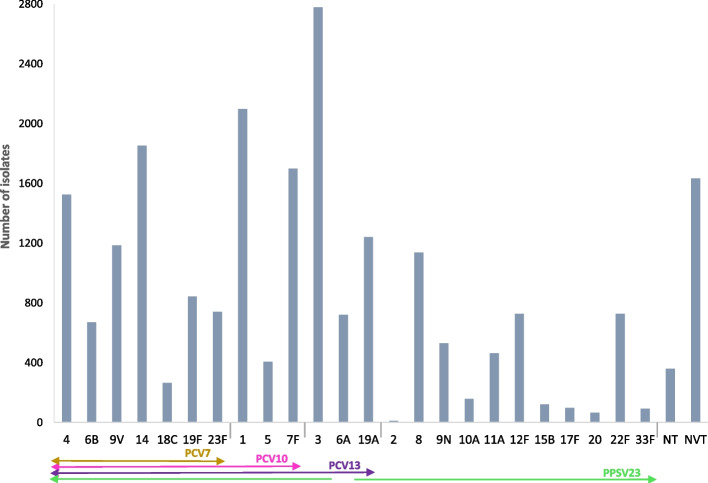
Distribution of CAP isolates in studies with information on serotypes (*N* = 40 studies, 23,235 isolated serotypes) in adults of all ages (> 14 years old). NVT – Non-vaccine serotype: serotypes not included in any pneumococcal vaccine. NT – Non-typeable: serotype information not determined by the table method. PPSV23 contains all vaccine types in PCV13 except serotype 6A

The distribution of the serotypes among pneumococcal pneumonia isolates from all 40 studies is presented in Fig. 3. Across all publications, the most common serotype causing CAP in adults (aged more than 14 years of age) was serotype 3 (2775 isolates, representing 11.9% of the total of isolates), followed by serotypes 1, 14, 7F, 4, 19A, 9V and 8. Serotype 2 had the lowest rate in all studies (< 0.1% in all age groups).

**Fig. 4:**
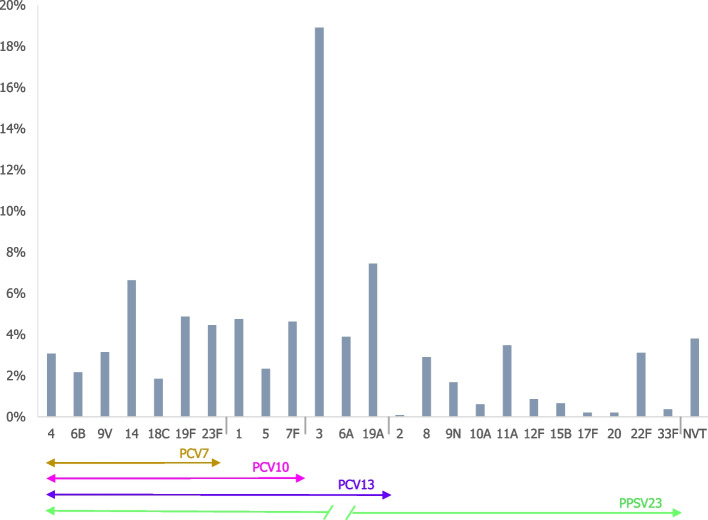
Distribution of pneumococcal pneumonia serotypes proportion in adults > 50 years of age (*N* = 12 studies, 2443 isolated serotypes) NVT – Non-vaccine type: serotypes not included in any pneumococcal vaccine

In adults aged more than 50 years, the most prevalent serotype was serotype 3 (18.9%), followed by serotypes 19A, and 14 (based on data of 12 publications for which a total of 2443 serotypes were available, Fig. 4).

### Distribution of serotypes according to the type of pneumonia (invasive or non-invasive)

**Fig. 5:**
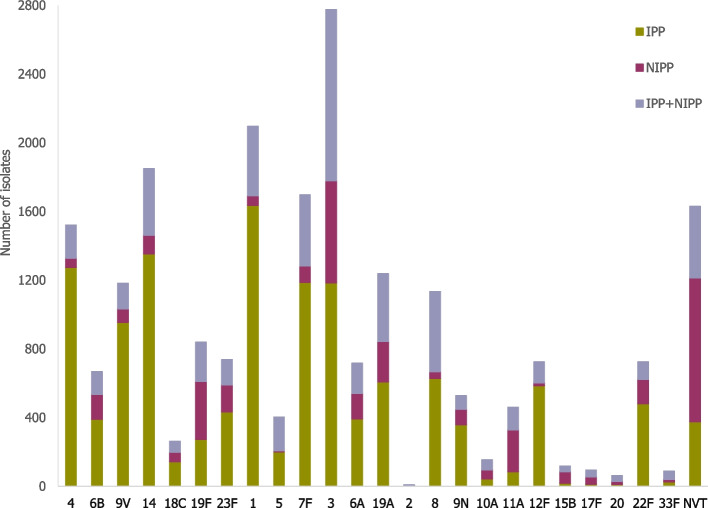
Distribution of serotypes according to the type of pneumonia. N_IPP_ = 12 studies^a^, 13,170 isolated serotypes—N_IPP_ = 9 studies^a^, 4227 isolated serotypes –N_IPP+NIPP_ = 22 studies, 5838 isolated serotypes—(*N* = 40 studies, 23,235 isolated serotypes) IPP: Invasive Pneumococcal Pneumonia NIPP: Non-invasive Pneumococcal Pneumonia ^a^Three studies distinguish serotypes isolated in IPP from NIPP in the results

In total, 13,170 isolates were from invasive pneumococcal isolates (IPP), 4227 from non-invasive pneumococcal isolates (NIPP) and 5838 from IPP + NIPP isolates (the study did not differentiate between serotypes from invasive and non-invasive pneumonia in the analysis) (Fig. 5).

### Proportion of non-vaccine serotypes (NVT)

**Fig. 6:**
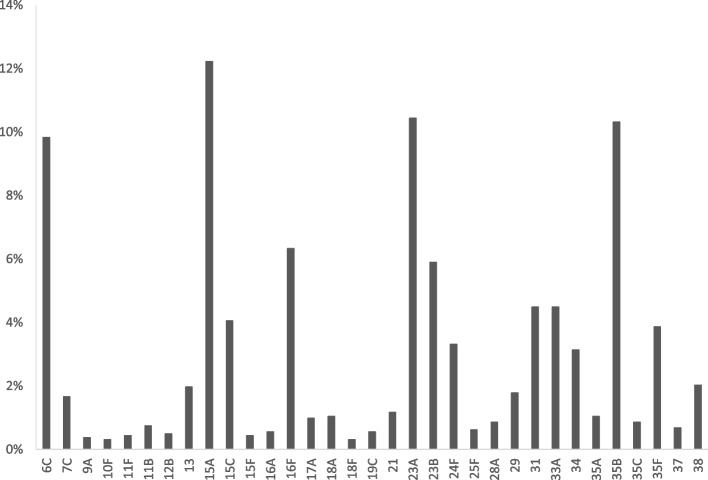
Proportion of NVT from all NVT isolates (*N* = 28 studies, 1629 isolated serotypes), adults of all age

Proportion of most common non-vaccine serotypes found in identified studies is represented in Fig. 6. The most common non-vaccine serotype in adults of all ages was serotype 15A, representing 12.2% from all NVT isolates (N_NVT_ = 1629), followed by serotypes 23A, 35B, 6C, 16F and 23B.

### Distribution of serotypes according to the PCV period

**Fig. 7:**
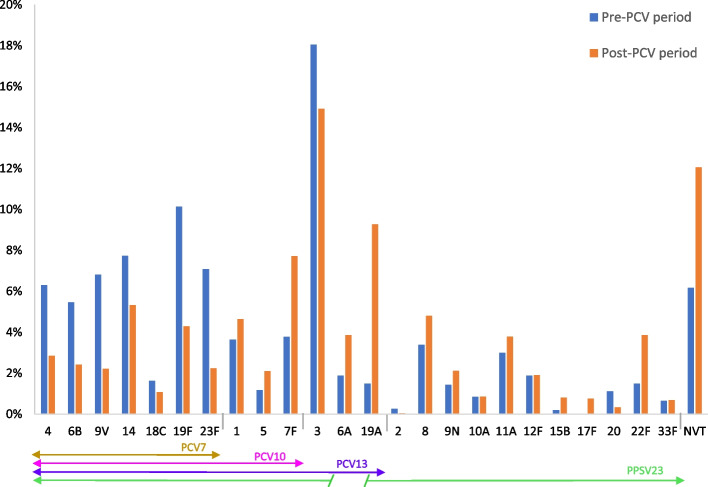
Proportion of serotypes before (*N* = 9 studies, 1540 isolated serotypes) and after (*N* = 25 studies, 10,452 isolated serotypes) PCVs introduction

**Fig. 8:**
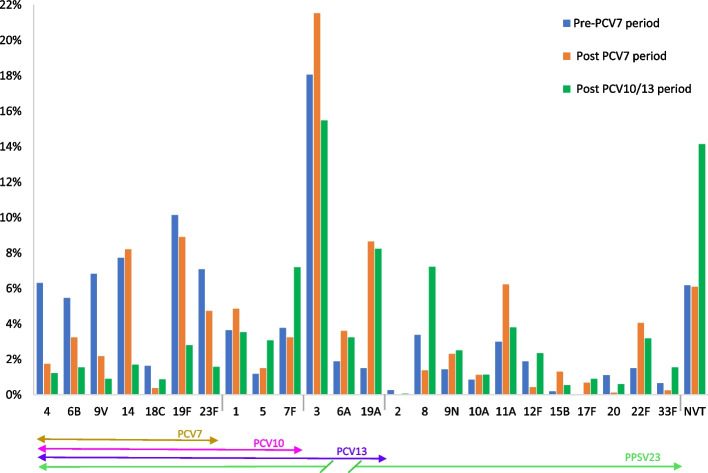
Proportion of serotypes according to the PCV period in adults of all age (N_ST_ Pre-PCV7 period = 1540 isolated serotypes (data from 9 studies)—N_ST_ Post-PCV7 period = 1608 isolated serotypes (data from 7 studies)—NST Post PCV10/13 period = 3367 isolated serotypes (data from 12 studies)) Serotype proportions were calculated by period (e.g., the proportion of a serotype for a specific period = number of a serotype in the period/ number total of ST in the period)

The distribution of serotypes according to the PCV period^2^ is represented in Figs. 7 and 8.

The proportion of serotype 3 isolates was higher for all periods and still represented 15.1% of serotypes in the post PCV10/13 period.

The post PCV period was marked by an increase of serotypes 19A, 11A, 22F and 6A, serotypes not included in PCV7.

**Fig. 9:**
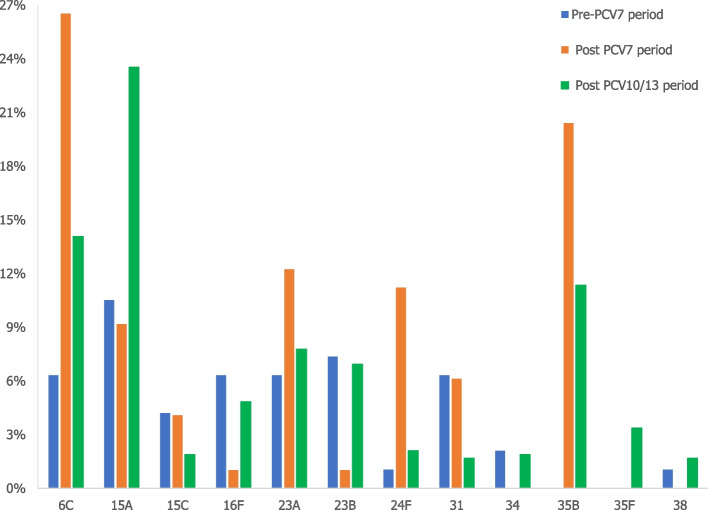
Distribution of most common NVT (from all NVT by period) in adults of all age (NVT_T_ Pre-PCV7 period = 95, NVT_T_ Post-PCV7 period = 98, NVT_T_ Post PCV10/13 period = 476). Note: Data from studies on the overlapping period were not included in Figs. 7, 8 and 9

In the PCV10/13 period, there was a decrease in PCV7 serotypes, but the frequency of serotypes 19A, 6A, 7F and 8 remained high despite their inclusion in a vaccine. The higher frequency of NVT is observed during this period (15.2%), with the most common NVT being serotype 15A, followed by serotypes 6C and 35B (Fig. 9).

The pre-PCV7 period includes studies conducted between 1977 and 2007, the post-PCV7 period covers studies conducted between 2001 and 2011, and the post-PCV10/13 period, studies conducted between 2009 and 2018.

### Proportion of pneumococcal CAP due to vaccine types

Serotypes of PPSV23 represented 83.3% of total CAP, following by PCV13 (68.8%), PCV10 (48.4%) and PCV7 serotypes (30.4%).

NVTs represented 7% of the total CAP isolates.

The proportion represented by the PPSV23 non-PCVs serotypes (i.e., serotypes included only in the PPSV23) was 17.6%. The 6 additional PCV13 serotypes had a proportion of 38.5%.

### Distribution of serotypes by vaccine period

**Fig. 10:**
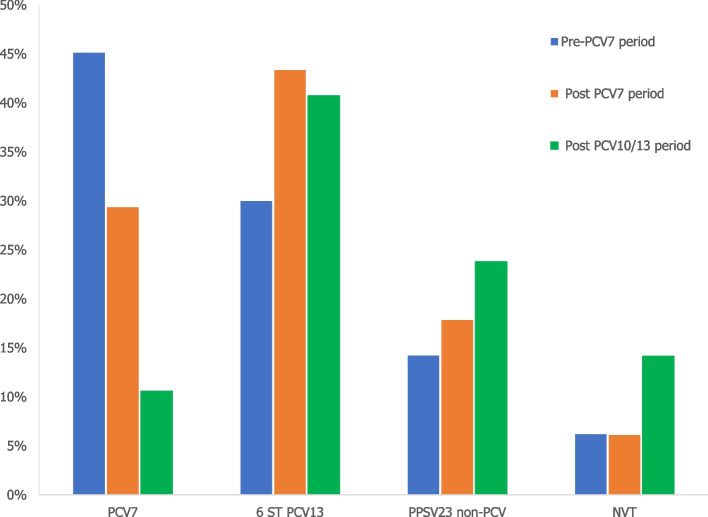
Proportion of serotypes causing PP according to the period. 6 ST additional of PCV13 = 1, 3, 5, 6A, 7F, 19A; PPSV23 non-PCV = 2, 8, 9N, 10A, 11A, 12F, 15B, 17F, 20, 22F, 33F)

The proportion of the six additional PCV13 serotypes decreased slightly between post-PCV7 and post-PCV10/13 periods (43.3% and 40.7% respectively) and the proportion of NVTs increased in the post PCV10/13 period (14% versus 6% in the previous periods) (Fig. 10).

### Distribution of serotypes by regions

The proportion of vaccine and non-vaccine serotypes causing pneumococcal pneumonia across regions is shown in Supplementary Table S6. Most of the studies included in this review were conducted in Europe (27 studies), 6 studies were conducted in America (4 publications in North America and 2 in Latina America) and 7 studies in Asia (including Australia).

**Fig. 11:**
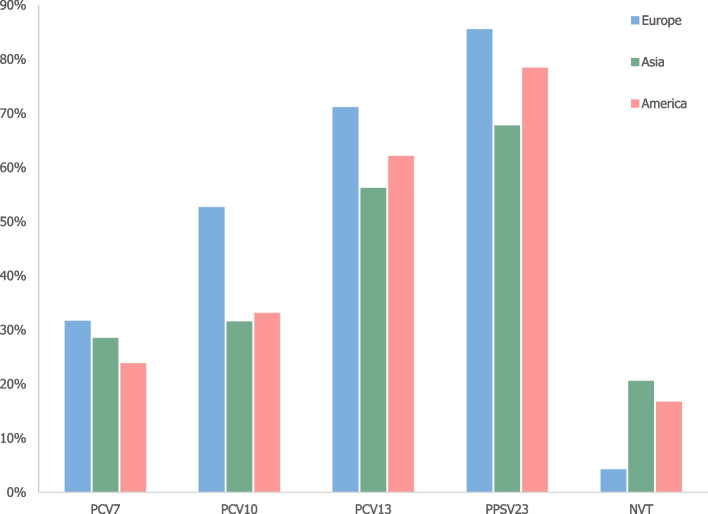
Proportion of vaccine and non-vaccine serotypes by regions

The proportion of non-vaccine serotypes was the lowest in Europe (4.1% against 20.5% and 16.6% in Asia and America respectively) (Fig. 11).

**Fig. 12:**
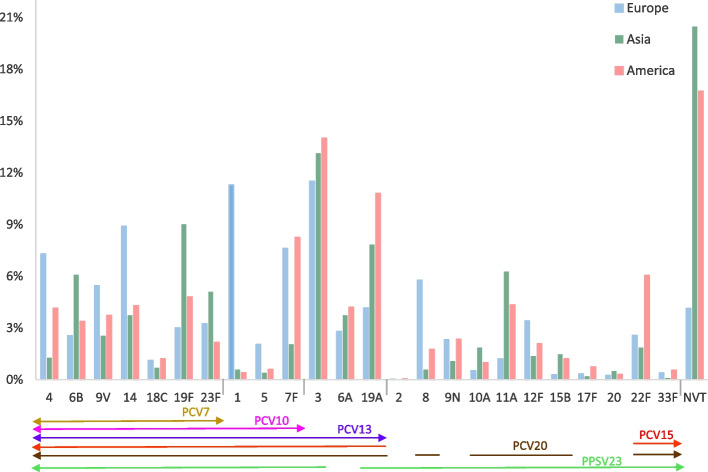
Proportion of pneumococcal CAP serotypes across regions

The distribution of serotypes by regions is illustrated in Fig. 12.

The results showed that some serotypes have a heterogeneous distribution across regions. As an example, while the proportion of serotype 1 was the highest in Europe (11.3% vs. 0.4% for the other regions), serotype 19F was the most frequent in the Asia region (9% vs. 3–4.8% in other regions).

All studies showed a high prevalence of serotype 3.

The most frequent NVT was 35B, followed by 6C and 15A (5%, 3.6% and 2.7% respectively in Asia).

## Discussion

In this review, we aimed to determine the most common serotypes causing pneumococcal pneumonia in the elderly. Serotype 3 was the leading serotype identified across studies and age groups, with a prevalence remaining high in the post PCV10/13 period. Serotypes 1, 14, 7F, 4, 19A, 9 V and 8 were the following serotypes. In adults > 50 years old, after serotypes 3, the most frequent serotypes were serotypes 19A and 14.

Globally, we observed a decreasing trend of PCV7 serotypes after its introduction in pediatric NIP which was offset by an increase of non-vaccine serotypes. This is in accordance with published data from different countries showing that following the introduction of PCV7, carriage of PCV7 serotypes in unvaccinated children and adults decreased with an increase in non-vaccine types [61–63]. The most frequent non-PCV7 serotype responsible for that increase was serotype 3, followed by serotypes 19A and 11A. This finding is also consistent with published literature [5, 18]. Some studies explain the high rate of serotypes 3 and 19A by their low immunogenicity, suggesting also an absence of herd protection [37, 64].

It has also been observed that the frequency of serotypes 3, 19A and 7F remained high even after the introduction of PCV10/13 into childhood immunization programs. Our study also showed an increase of serotype 8 (included in PPSV23) in the PCV10/13 period.

Consistent with previous publications, our study suggests that serotypes 3, 7F and 19A still cause disease and remain the most common serotypes among adults with pneumococcal pneumonia, despite the availability of pneumococcal vaccines covering those serotypes [17, 65–67].

We observed a heterogeneous distribution of some serotypes according to geographical region. For example, the proportion of pneumococcal CAP due to serotypes 1, 14 and 8 was higher in Europe (11.2%, 9% and 6% respectively versus 0.4%, 3.7–4.3% and 0.6–1.8% in other regions).

Previous publications have also reported an increase in serotype 8 in adult pneumococcal pneumonia in Europe, but in other countries, such as the United-States, no increase has been described [68].

It should be nevertheless noted that despite the availability of pneumococcal vaccines (PPSV23 or PCVs), that does not mean that the vaccines are included in national immunization programs nor that they effectively used by this age group.

Furthermore, as shown in a multicenter European study that estimated the indirect effects of a 5-year childhood PCV10 and/or PCV13 programs on IPD in older adults, the decrease of vaccine-types due to the indirect effect of childhood vaccination was offset by the increase of non-PCV13 serotypes [69].

Our study has shown that the proportion of the non-vaccine serotypes in pneumococcal pneumonia increased after introduction of PCVs.

Non-vaccine serotypes represented 7% of the total number of serotypes causing pneumococcal pneumonia in the studies included in this review. Among NVTs, the most common serotype in all age groups, was serotype 15A, followed by serotypes 23A, 35B, 6C, 16F and 23B. In adults > 50 years of age, the most common NVTs were serotypes 6C, 24F, 35B and 31.

Thus, most of pneumococcal pneumonia was due to vaccine serotypes. Overall, 83% of serotypes causing pneumococcal CAP were covered by PPSV23 and almost 69% by PCV13 (of which 38% by the six additional serotypes). During the PCV10/13 period, we observed a decline of pneumococcal pneumonia due to PCV7 serotypes (10% versus 45% in PCV7 period), but PCV13 serotypes (51%, of which 41% attributable to the six additional serotypes) remains high.

Across the development of pneumococcal conjugated vaccines’ history, the number of serotypes included in the vaccines have increased from 7 serotypes in PCV7 to 13 serotypes in PCV13 vaccine. In 2021, two new vaccines were approved by the Food and Drug Administration (FDA) for the prevention of invasive disease caused by *S. pneumoniae*: PCV15 and PCV20.

The PCV15 is indicated for active immunization for the prevention of invasive disease caused by *S. pneumoniae* in individuals 6 weeks of age and older [70].

The PCV20 is recommended for active immunization for the prevention of pneumonia (by serotypes 8, 10A, 11A, 12F, 15B, 22F, and 33F) and invasive disease caused by *S. pneumoniae* in adults 18 years of age and older [71].

The PCV15 and PCV20 vaccines include PCV13 serotypes plus some serotypes included in PPSV23 (22F, 33F, and 8, 10A, 11A, 12F, 15B, 22F, and 33F respectively) [72]. Increasing vaccine valency may increase non-vaccine serotypes, and it is difficult to predict how the composition of NVTs will change after the introduction of a new PCV [73].

On October 2021, the ACIP recommended use of PCV20 alone or PCV15 in series with PPSV23 for PCV–naïve adults who are either aged ≥ 65 years or aged 19–64 years with certain underlying conditions [14].

Future studies evaluating the effectiveness and impact of the introduction of these new vaccines may support the guidance of further recommendation policies.

New approaches to design an effective pneumococcal vaccine are underway in response to the worldwide pneumococcal burden. New pneumococcal vaccines must take into consideration, among others, the local epidemiology and burden of disease in different regions of the world, target age groups, the different disease entities as well as the emergence of non-vaccine serotypes.

Our study has several limitations. It is a descriptive review based only on 40 studies, so the distribution of serotypes may be not representative of the global burden. A very limited number of publications reported specifically on serotypes causing pneumococcal pneumonia in the elderly. To overcome this, we have chosen to report the available information from studies including other age groups provided that they also included the elderly population. Most published literature reported on IPD and/or focus on children and lacked information on serotype distribution by targeted age group or disease entity. Investigation and publication in this area should be encouraged. Moreover, there is a lack of data on the impact of vaccination programs in the elderly on pneumococcal pneumonia.

Sixty-seven percent of studies included (27 studies) were conducted in Europe (with one study covering 1/3 of all data), which could have biased the results towards guidelines implemented in this region and may not be representative of the global distribution of CAP serotypes.

Sero-epidemiological data from low-income countries are lacking.

It should also be noted that there are differences in the timing of vaccine introduction, vaccination policies and vaccine coverage among countries that implemented PPVS23 and PCVs into their National Immunization Programs (NIPs). This could result in differences in direct and indirect effects of the vaccines as well as in serotypes circulating [66, 74], which could difficult the interpretation of the results.

A diagnostic bias cannot be excluded either. In fact, the serotypes detected in surveillance studies will depend on the methods used for diagnosis. Some studies showed that the serotyping method used was only able to detect some serotypes (e.g. aim to assess or compare serotyping methods) [52]. Therefore, the proportion of some serotypes could have been over or under-reported due to the serotyping method used. As an example, studies using PCV13-specific urine antigen detection could have led to an underrepresentation of PPSV23 serotypes (non-PCV13) and the proportion of non-typeable serotypes could have been overrepresented [23, 25, 26, 43, 55, 57, 60]. In addition, some studies have reported cross-reactivity between serotypes due to the serotyping method used (e.g., BioPlex). Also, some authors chose to classify untyped serotypes as NVT, which can lead to an over-representation of NVTs [41].

This review showed that the most frequent serotypes responsible for pneumococcal disease in adults are included in existing vaccines (PCVs and PPSV23), suggesting that either the vaccines are not as effective in this population as in other age groups or that the coverage of the vaccine is sub-optimal in this population. Patients aged > 65 years are especially vulnerable to pneumonia because of both age-related changes in the immune system and of a greater prevalence of chronic diseases, and should, therefore, be a primary target of vaccination programs [75].

Our study highlights the importance to monitor serotypes causing pneumococcal pneumonia in adults as well as to monitor the evolution of serotype replacement to better target vaccination programs and support the development of new vaccines.

## Conclusion

The results of this study showed that some of the most frequent serotypes causing pneumococcal pneumonia in adults are vaccine serotypes. Inadequate protection against some capsular serotypes (e.g., serotype 3), low coverage of the vaccines in this age group and serotype replacement, are some factors to consider.

The study highlights the importance of surveillance and monitoring of disease-causing serotypes emerging in this population to better target vaccination strategies.

## Supplementary Information

Supplementary Material 1

## Data Availability

Individual participants’ data that underlie the results reported in this article and a data dictionary defining each field in the set are available to investigators whose proposed use of the data has been approved by an independent review committee for work. Proposals should be directed to weima@sdu.edu.cn to gain access, data requestors will need to sign a data access agreement. Such requests are decided on a case by case basis.

## Abbreviations

ACIP: Advisory Committee on Immunization Practices
CAP: Community-acquired pneumonia
IPD: Invasive pneumococcal disease
IPP: Invasive pneumococcal pneumonia
NIP: National Immunization Program
NIPP: Non-invasive pneumococcal pneumonia
NVT: Non-vaccine serotypes
PCVs: Pneumococcal conjugate vaccines
PCV7: Heptavalent Pneumococcal Conjugate Vaccine
PCV10: Ten-valent Pneumococcal Conjugate Vaccine
PCV13: Thirteen-valent Pneumococcal Conjugate Vaccine
PPSV23: Pneumococcal polysaccharide-based vaccine
ST: Serotype

## Footnotes

1 Studies with population from 14—16 years of age were included if the original study did not discriminate the age groups but elderly population was included.

2 PCV periods were defined based on the pediatric NIP.
