# Supplementary material for: Systematic review on serotypes distribution of pneumococcal pneumonia in adults and the elderly
Source: BMC Public Health. 2025 Mar 29;25:1194. doi: 10.1186/s12889-025-22164-x (PMC11954302; doi:10.1186/s12889-025-22164-x)
Supplement: Supplementary file 1 — Supplementary Material 1 [file 12889_2025_22164_MOESM1_ESM.docx]

**S1**

**Search strategy**

|  | **SEARCH TERMS AND COMBINATIONS** | **RESULTS** |
| --- | --- | --- |
| 1 | Streptococcus pneumoniae [Mesh] | 22 894 |
| 2 | Streptococcus pneumoniae [Text Word] | 33 761 |
| 3 | S. pneumoniae [Text Word] | 9 857 |
| 4 | ((Streptococcus pneumoniae [Mesh]) OR (streptococcus pneumoniae [Text Word])) OR (s. pneumoniae [Text Word]) | 34 795 |
| 5 | Pneumococcal Infections [Mesh] | 20 821 |
| 6 | Pneumococcal Infections [Text Word] | 14 001 |
| 7  #5 OR #6 | (Pneumococcal Infections [Mesh]) OR (Pneumococcal Infections [Text Word]) | 21 269 |
| 8  #4 OR #7 | (((Streptococcus pneumoniae [Mesh]) OR (streptococcus pneumoniae [Text Word])) OR (s. pneumoniae [Text Word])) OR ((Pneumococcal Infections [Mesh]) OR (Pneumococcal Infections [Text Word])) | 42 642 |
| 9 | Pneumonia [Mesh] | 128 894 |
| 10 | Pneumonia, Pneumococcal [Mesh] | 5 186 |
| 11 | Pneumonia, Pneumococcal [Text Word] | 5 226 |
| 12 | Pneumococcal Pneumonia [Text Word] | 2 257 |
| 13  #9 OR #10 OR #11 OR #12 | (((Pneumonia [Mesh]) OR (Pneumonia, Pneumococcal [Mesh])) OR (Pneumonia, Pneumococcal [Text Word])) OR (Pneumococcal Pneumonia [Text Word]) | 129 490 |
| 14 | Community-Acquired Infections [Mesh] | 14 431 |
| 15 | Community-Acquired Infections [Text Word] | 15 357 |
| 16 | Community-Acquired Pneumonia [Text Word] | 10 158 |
| 17  #14 OR #15  OR #16 | ((Community-Acquired Infections [Mesh]) OR (Community-Acquired Infections [Text Word])) OR (Community-Acquired Pneumonia [Text Word]) | 19 187 |
| 18  #13 OR #17 | ((((Pneumonia [Mesh]) OR (Pneumonia, Pneumococcal [Mesh])) OR (Pneumonia, Pneumococcal [Text Word])) OR (Pneumococcal Pneumonia [Text Word])) OR (((Community-Acquired Infections Mesh]) OR (Community-Acquired Infections [Text Word])) OR (Community-Acquired Pneumonia [Text Word])) | 139 637 |
| 19  #8 AND #18 | ((((Streptococcus pneumoniae [Mesh]) OR (streptococcus pneumoniae [Text Word])) OR (s. pneumoniae [Text Word])) OR ((Pneumococcal Infections [Mesh]) OR (Pneumococcal Infections [Text Word]))) AND (((((Pneumonia [Mesh]) OR (Pneumonia, Pneumococcal [Mesh])) OR (Pneumonia, Pneumococcal [Text Word])) OR (Pneumococcal Pneumonia [Text Word])) OR (((Community-Acquired Infections [Mesh]) OR (Community-Acquired Infections [Text Word])) OR (Community-Acquired Pneumonia [Text Word]))) | 9 126 |
| 20 | Serotype [Text Word] | 35 973 |
| 21 | ST [Text Word] | 112 511 |
| 22 | non-PCV* [Text Word] | 380 |
| 23  #20 OR #21 | (Serotype [Text Word]) OR (ST [Text Word]) | 147 861 |
| 24  #22 OR #23 | ((Serotype [Text Word]) OR (ST [Text Word])) OR (non-PCV* [Text Word]) | 147 959 |
| 25  #19 AND #24 | ((((("Streptococcus pneumoniae"[Mesh]) OR (streptococcus pneumoniae[Text Word])) OR (s. pneumoniae[Text Word])) OR (("Pneumococcal Infections"[Mesh]) OR (Pneumococcal Infections[Text Word]))) AND ((((("Pneumonia"[Mesh]) OR ("Pneumonia, Pneumococcal"[Mesh])) OR (Pneumonia, Pneumococcal[Text Word])) OR (Pneumococcal Pneumonia[Text Word])) OR ((("Community-Acquired Infections"[Mesh]) OR (Community-Acquired Infections[Text Word])) OR (Community-Acquired Pneumonia[Text Word])))) AND (((serotype[Text Word]) OR (ST[Text Word])) OR (non-PCV*[Text Word])) | 686 |
| 26  #25 NOT Children | (((((("Streptococcus pneumoniae"[Mesh]) OR (streptococcus pneumoniae[Text Word])) OR (s. pneumoniae[Text Word])) OR (("Pneumococcal Infections"[Mesh]) OR (Pneumococcal Infections[Text Word]))) AND ((((("Pneumonia"[Mesh]) OR ("Pneumonia, Pneumococcal"[Mesh])) OR (Pneumonia, Pneumococcal[Text Word])) OR (Pneumococcal Pneumonia[Text Word])) OR ((("Community-Acquired Infections"[Mesh]) OR (Community-Acquired Infections[Text Word])) OR (Community-Acquired Pneumonia[Text Word])))) AND (((serotype[Text Word]) OR (ST[Text Word])) OR (non-PCV*[Text Word]))) NOT (children[Text Word]) | 413 |

| 27  #26 + Filters : “Humans” , “from 1984 - 2020” | **306** |
| --- | --- |

**S2**

**Characteristics of excluded studies**

| STUDY | REASON OF EXCLUSION |
| --- | --- |
| Hauser, 2016 | Wrong population (children) |
| Brueggemann, 2007 | Wrong disease (IPD) / Wrong population (children) |
| Ortqvist, 2007 | Wrong outcomes |
| Song, 2014 | Wrong population |
| Song, 2013 | Wrong study design (Review) |
| Chalmers, 2016 | Wrong study design (Systematic review) |
| Cohen, 2015 | Wrong disease (IPD) |
| Musher, 1997 | Wrong outcomes |
| Wantuch, 2018 | Wrong outcomes |
| Martens, 2004 | Wrong disease (IPD) |
| Wagenvoort GHJ, 2017 | Wrong outcomes |
| LeBlanc, 2017 | Wrong outcomes |
| MedeirosMIC, 2017 | Wrong population |
| Dicuonzo, 2002 | Wrong outcomes |
| Skoczyńska, 2012 | Wrong outcomes |
| Scott, 2000 | Wrong outcomes |
| Schroeder, 2017 | Wrong outcomes |
| Isea-Peña, 2013 | Wrong disease |
| Inghammar, 2018 | Wrong population |
| Orsi, 2016 | Wrong outcomes |
| Pankuch, 2002 | Wrong outcomes |
| Thorrington, 2018 | Wrong outcomes |
| Mangtani, 2003 | Wrong study design (Meta-analysis) |
| Chen, 2017 | Wrong population |
| Imai, 2009 | Wrong outcomes |
| Weinberger, 2010 | Wrong study design (meta-analysis) |
| Moffitt, 2011 | Wrong outcomes |
| Vainio, 2009 | Wrong outcomes |
| Klugman, 2009 | Wrong population |
| Plosker, 2015 | Wrong study design (Review) |
| Holliman, 2007 | Wrong population |
| McNabb, 1984 | Wrong outcomes |
| Casal, 1982 | Wrong outcomes |
| Fouda, 2004 | Wrong outcomes |
| Alnimr, 2017 | Wrong population |
| Farrell, 2008 | Wrong population |
| Zhanel, 2018 | Wrong outcomes |
| Li, 2019 | Wrong disease |
| Jayaraman, 2019 | Wrong outcomes |
| Nuorti, 1998 | Wrong outcomes |
| Gleich, 2000 | Wrong outcomes |
| Prebil, 2016 | Wrong outcomes |
| Einarsson, 1998 | Wrong outcomes |
| Domenech, 2014 | Wrong disease / outcomes |

**S3**

**General characteristics of the studies included in the systematic review**

| **Study** | **Country** | **Year** | **Design** | **Sample**  **(PP)** | **Age (y)**  **Median (m) or Mean age (m)** | **Most prevalent serotypes** | **Vaccinated**  **(%)** | **Vaccine product** |
| --- | --- | --- | --- | --- | --- | --- | --- | --- |
| Shigayeva (19) | Canada | 2003-2011 | Population-based surveillance | 2060 BPP^[[1]](#footnote-1)^  1542 NBPP^[[2]](#footnote-2)^ | > 15  M = 63 | 19A, 3, 7F  3, 19F, 11A | 32.5  39.7 | PPSV23 |
| Maraki (20) | Greece | 2001-2008 |  | 195 | > 19 | 19F, 6B, 3, 19A |  |  |
| Sherwin (75) | US | 2010-2011 | Prospective, cross-sectional, multicenter study | 98  CAP+HCAP^[[3]](#footnote-3)^ | ≥ 50  M = 62.2 | 19A, 7F/A, 3, 5 | 40.8 | PPSV23 |
| Jacups (22) | Australia | 1987-2008 | Prospective + Retrospective cohorts | 197 | > 14  M = 40 | 7F, 19A, 4, 14, 12F, 18A |  |  |

| **Study** | **Country** | **Year** | **Design** | **Sample**  **(PP)** | **Age (y)**  **Median (m) or Mean age (m)** | **Most prevalent serotypes** | **Vaccinated**  **(%)** | **Vaccine product** |
| --- | --- | --- | --- | --- | --- | --- | --- | --- |
| VanWerkhoven (23) | Netherlands | 2008-2013 | Prospective study | 288 | > 65 | 3, 19A, 7F |  |  |
| Rodrigo (24) | UK | 2008-2013 | Prospective cohort study | 653 | > 16  M = 69.2 | 1, 7F/A, 19A | 55.6 | PPSV23 |
| Domenech (25) | Spain | 2001-2008 | Laboratory-based study | 255 | ≥ 50  m = 70 | 3, 1, 5 (BP)  3, 19F, 23F, 11A (NBP) |  |  |
| Forrester (26) | US | 1979-1985 | Case-control study | 60 | ≥ 50  m = 64.7 | 3, 4, 9, 14, 19, 23 | 29 | 14-valent PV |
| Morgan (27) | Scotland | 1978-1983 | Retrospective study | 125 | > 15  m = 64 | 3, 4, 7 |  |  |
| Cardinal-Fernandez (28) | Spain | 2008-2010 | Prospective, observational, cohort study | 192 | > 16  m = 54.6 | 7F, 1, 3 | 10 (1 year before)  4 (5 year before) | PPSV23 |

| **Study** | **Country** | **Year** | **Design** | **Sample**  **(PP)** | **Age (y)**  **Median (m) or Mean age (m)** | **Most prevalent serotypes** | **Vaccinated**  **(%)** | **Vaccine product** |
| --- | --- | --- | --- | --- | --- | --- | --- | --- |
| Fica (29) | Chile | 2005-2010 | Retrospective and descriptive study | 22 | > 18  M = 72.1 | 14, 7F, 9V, 12F, 22F, 6A, 8, 4, 1 |  |  |
| Oishi (30) | Japan | 2001-2003 | Nationwide prospective study | 114 | > 16  m = 67.4 | 19F, 23F, 6B, 3 |  |  |
| Akata (31) | Japan | 2011-2013 | Retrospective epidemiological study | 81  (CAP, HCAP^[[4]](#footnote-4)^, HAP^[[5]](#footnote-5)^) | m = 66.7 | 3, 6A/B, 11A/E | 12.4% | PPSV23 |
| Horacio (32) | Portugal | 1999-2011 | Laboratory-based surveillance | 1300 | > 18 | 3, 11A, 19F, 19A |  |  |
| Perez-Trallero (33) | Spain | 2005-2008 | Prospective laboratory-based study | 394 | > 65  m = 77.1 | 3, 19A, 14, 7F |  |  |

| **Study** | **Country** | **Year** | **Design** | **Sample**  **(PP)** | **Age (y)**  **Median (m) or Mean age (m)** | **Most prevalent serotypes** | **Vaccinated**  **(%)** | **Vaccine product** |
| --- | --- | --- | --- | --- | --- | --- | --- | --- |
| Aspa (34) | Spain | 1999-2000 | Multicenter observational prospective study | 638 | > 16  m = 61.5 | 3, 19, 14 |  |  |
| Echaniz-Aviles (76) | Mexico | 2000-2015 | Retrospective study | 66 | > 18  M = 43 | 19A, 3, 23F |  |  |
| Ortqvist (36) | Sweden | 1977-1984 | Retrospective study | 279 | > 15  m = 56 | 3, 9, 4 |  |  |
| Qin (37) | Japan | 2001-2003 | Prospective study | 114 | > 20  m = 67.4 | 3, 6B, 19F |  |  |
| Wattal (38) | India | 2013-2015 | Prospective surveillance study | 50 | ≥ 50 | 19A, 8, 19F, 3, 9N |  |  |
| Pick (77) | UK | 2013-2018 | Population-based  prospective cohort study | 1075 | > 16  M = 69.3 | 3, 8, 15A |  |  |

| **Study** | **Country** | **Year** | **Design** | **Sample**  **(PP)** | **Age (y)**  **Median (m) or Mean age (m)** | **Most prevalent serotypes** | **Vaccinated**  **(%)** | **Vaccine product** |
| --- | --- | --- | --- | --- | --- | --- | --- | --- |
| Sando (40) | Japan | 2011-2014  2016-2017 | Multicentre prospective study  (2 phases) | 223  215 | > 15  M = 73 | 3, 11A, 19A, 19F  35B, 3, 19A, 11A | ≈ 1%  19%^[[6]](#footnote-6)^  3% ^[[7]](#footnote-7)^ | PCV13  PSV23 |
| Bewick (41) | UK | 2008-2010 | Prospective observational cohort study | 366 | > 16  M = 71.1 | 14, 1, 8, 3, 19A |  |  |
| Vallès (42) | Spain | 1999-2002 | Prospective study | 125 | > 16  m = 59.6 | 3, 1, 14, 6B |  |  |
| Lujan (43) | Spain | 1996-2011 | Observational study | 1094 | > 18  m = 60.9 | 1, 3, 14, 7F |  |  |
| Vila-Corcoles (44) | Spain | 2008-2011 | Prospective study | 125 | > 60  m = 71.1 | 3, 19A,6C,22F, 35B |  |  |

| **Study** | **Country** | **Year** | **Design** | **Sample**  **(PP)** | **Age (y)**  **Median (m) or Mean age (m)** | **Most prevalent serotypes** | | **Vaccinated**  **(%)** | | **Vaccine product** |
| --- | --- | --- | --- | --- | --- | --- | --- | --- | --- | --- |
| Lujan (45) | Spain | 1999-2009 | Prospective, 10-yr observational study | 299 | > 18  m = 62.2 | 1, 3, 14 | 20.7% | | PPSV23 | |
| Cilloniz (46) | Spain | 2001-2009 | Prospective study | 626 | m = 63.6 | 1, 19A, 3, 14, 7F, 5 |  | |  | |
| Harat (47) | Poland | 2010-2012 | Prospective population-based surveillance study | 144 | > 50  m = 68.3 | 3, 23F, 18C, 9V |  | |  | |
| Choi (48) | South Korea | 2015 | Retrospective study | 92 | > 15  m = 66.1 | 19A, 3, 11A, 34, 35B, 23A, 10A, 19F | | 29.3%  7.6% | | PPSV23  PCV13 |
| Benfield (49) | Denmark | 2011 | Population-based design | 192 BP  272 NBP | > 15  M = 68 | 1, 7F, 3, 8, 19A  3, 11A, 19A, 6C, 7F |  | |  | |

| **Study** | **Country** | **Year** | **Design** | **Sample**  **(PP)** | **Age (y)**  **Median (m) or Mean age (m)** | **Most prevalent serotypes** | **Vaccinated**  **(%)** | **Vaccine product** |
| --- | --- | --- | --- | --- | --- | --- | --- | --- |
| Elberse (50) | Netherlands | 2007-2010 | Samples were obtained from hospitalized CAP patients who participated in a trial | 278 | > 18  m = 63 | 3, 8, 1 |  |  |
| Garcia-Vidal (51) | Spain | 1995-2008 | Prospective study | 1041 | > 18  M = 70 | 3, 1, 14 | 14% | PPSV23 |
| Weinberger (52) | Denmark | 1977-2007 | Danish National Laboratory Surveillance System | 9941 | > 40 | 1, 14, 4 |  |  |
| Rodrigo (53) | UK | 2008-2011 | Observational prospective cohort study | 415 | > 16  M = 68.2 | 14, 1, 8 |  |  |
| Bedos (54) | France | 2008-2012 | Multicenter prospective study | 614 | > 18  M = 63 | 3, 7F, 19A, 12F, 1, 6C, 11A |  |  |

| **Study** | **Country** | **Year** | **Design** | **Sample**  **(PP)** | **Age (y)**  **Median (m) or Mean age (m)** | **Most prevalent serotypes** | **Vaccinated**  **(%)** | **Vaccine product** |
| --- | --- | --- | --- | --- | --- | --- | --- | --- |
| LeBlanc (55) | Canada | 2014-2015 | Active surveillance | 286 | > 16  m = 62.2 | 3, 7F, 19A | 56.8% | PCV13/PPSV23 |
| Naucler (56) | Sweden | 2007-2009 | Population-based cohort study | 1580 | > 18 | 18C, 9V, 7F, 4, 3 |  |  |
| Athlin (57) | Sweden | 1999-2002 | Prospective study | 70 | > 18  m = 66.3 | 3, 7F, 14, 23F |  |  |
| Daniel (58) | UK | 2008-2013 | Prospective cohort study | 643 | > 16 | 7F/A, 8, 1 | 45.3 % | PPSV23 |

**S4**

**Type of pneumococcal pneumonia in studies included**

| **Invasive Pneumococcal Pneumonia + Non-Invasive Pneumococcal Pneumonia (IPP+NIPP)** | | |
| --- | --- | --- |
| Shigayeva^[[8]](#footnote-8)^ (19) | Wattal (38) | Elberse (50) |
| Sherwin (75) | Pick (77) | Garcia-Vidal (51) |
| Rodrigo (24) | Sando (40) | Rodrigo (53) |
| Domenech^1^ (25) | Bewick (41) | Bedos (54) |
| Morgan (27) | Vallès (42) | LeBlanc (55) |
| Cardinal-Fernandez (28) | Vila-Corcoles (44) | Athlin (57) |
| Oishi (30) | Harat (47) | Daniel (58) |
| Perez-Trallero (33) | Choi (48) |  |
| Aspa (34) | Benfield^1^ (49) |  |

| **Non-Invasive Pneumococcal Pneumonia**  **(NIPP)** | **Invasive Pneumococcal Pneumonia**  **(IPP)** |
| --- | --- |
| Maraki (20) | Jacups (22) |
| VanWerkhoven (23) | Forrester (26) |
| Akata (31) | Fica (29) |
| Horacio (32) | Ortqvist (36) |
| Echaniz-Aviles (76) | Lujan (43) |
| Qin (37)^[[9]](#footnote-9)^ | Lujan (45) |
|  | Cilloniz (46)^[[10]](#footnote-10)^ |
|  | Weinberger (52) |
|  | Naucler (56) |

**S5**

**Pneumococcal Vaccine Recommendations in children (countries included in this study)**

|  | **COUNTRY** | **PCV7** | | **PCV10** | **PCV13** | |
| --- | --- | --- | --- | --- | --- | --- |
|  |  | **Introduction** | **National Immunization Program (NIP)** |  |  |  |
| AMERICA | CANADA | June 2001 | 2005 | 2009 | 2010  (Replaced PCV10) | |
|  | CHILE | 2008^[[11]](#footnote-11)^ |  | 2011 | 2016^[[12]](#footnote-12)^ | |
|  | MEXICO | 2006^[[13]](#footnote-13)^ | 2008 |  | 2011 | |
|  | UNITED STATES |  | 2000 |  | 2010 | |
|  | **COUNTRY** | **PCV7** | | **PCV10** | | **PCV13** |
|  |  | **Introduction** | **National Immunization Program (NIP)** |  |  |  |
| ASIA | AUSTRALIA | 2001^[[14]](#footnote-14)^ | January 2005 | 2012 | | |
|  | INDIA | 2006^[[15]](#footnote-15)^ |  | 2010^5^  June 2017^[[16]](#footnote-16)^  2021^[[17]](#footnote-17)^ | | |
|  | JAPAN | October 2009 | 2010 |  | November 2013 | |
|  | SOUTH KOREA | November 2003 |  | March 2010  NIP in May 2014 | | |

|  | **COUNTRY** | **PCV7** | | **PCV10** | **PCV13** |
| --- | --- | --- | --- | --- | --- |
|  |  | **Introduction** | **National Immunization Program (NIP)** |  |  |
| EUROPE | DENMARK | 2001^1^ | October 2007 |  | April 2010 |
|  | FRANCE | 2003^6^ | June 2006 |  | June 2010 |
|  | GREECE |  | January 2006 | 2009 | 2010  (Replaced PCV10) |
|  | NETHERLANDS |  | June 2006 | April 2011 |  |
|  | POLAND |  |  | 2009 | 2010 |
|  |  |  |  | NIP in 2017^[[18]](#footnote-18)^ | |

|  | **COUNTRY** | **PCV7** | | **PCV10** | **PCV13** |
| --- | --- | --- | --- | --- | --- |
|  |  | **Introduction** | **National Immunization Program (NIP)** |  |  |
| EUROPE | PORTUGAL | 2001^[[19]](#footnote-19)^ |  | 2009 | 2010^[[20]](#footnote-20)^  NIP in 2015 |
|  | SPAIN  (Madrid)^[[21]](#footnote-21)^ | 2001 | November 2006 |  | June 2010 |
|  | SWEDEN | 2007^6^ | January 2009 | 2010 | |
|  | UK | 2002^1^ | September 2006 |  | April 2010 |

**S6**

**Percentage of Pneumococcal CAP caused by vaccine- and non-vaccine serotypes**

**Europe**

| Country | Period | Age, y | Other information | PCV7 serotypes  (%) | PCV10 serotypes  (%) | PCV13 serotypes  (%) | PPSV23 serotypes  (%) | NVTs  (%) |
| --- | --- | --- | --- | --- | --- | --- | --- | --- |
| Denmark (49) | 2011 | > 15 | NBPP  BPP |  | 16  40 | 34  59 | 57  87 |  |
| France (54) | 2008-2012 | > 18 | PCAP |  |  | 69.6 |  |  |
| Greece (20) | 2001-2008 | > 19 | Invasive pneumococcal isolates | 70.7 |  |  |  |  |
| Netherlands (23) | 2008-2013 | > 65 | PCAP^[[22]](#footnote-22)^ | 20 | 19 | 28 |  |  |
| Poland (47) | 2010-2012 | > 50 | CAP | 34 | 52.7 |  |  |  |
| Portugal (32) | 2009-2011 | > 18 | NIPP | 10.3 |  | 43.7 | 66 |  |

| Country | Period | Age, y | Other information | | | PCV7 serotypes  (%) | | PCV10 serotypes  (%) | | PCV13 serotypes  (%) | PPSV23 serotypes  (%) | NVTs  (%) |
| --- | --- | --- | --- | --- | --- | --- | --- | --- | --- | --- | --- | --- |
| Spain (28) | 2008-2010 | > 16 | CAP | | |  | |  | |  | 93 |  |
| Spain (33) | 2005-2008 | > 65 | Pneumonia | | | 19.5 | |  | | 63.3 | 75.1 |  |
| Spain (78) | 2008-2011 | > 60 | CAP | | |  | |  | | 53.6 | 73.1 |  |
| Spain (43) | 1996-2011 | > 18 |  | | | 24.7  (1996-2011) | |  | | 62  (1996-2011)  66  (2003-2011) |  |  |
| Spain (34) | 1999-2000 | > 16 | CAP | | |  | |  | |  | 88 |  |
| Spain (45) | 1999-2009 | > 18  < 60  > 60 | CAP | | |  | |  | |  | 91  94.1  87.8 |  |
| Spain (46) | 2006-2009 |  | PCAP | | | 13 | |  | |  |  |  |
| Spain (25) | 2001-2008 |  | BP + NBP | | | 28.2 | | 40.8 | | 65.1 | 79.6 |  |
| Country | Period | Age, y | | Other information | PCV7 serotypes  (%) | | PCV10 serotypes  (%) | | PCV13 serotypes  (%) | | PPSV23 serotypes  (%) | NVTs  (%) |
| Spain (53) | 2008-2011 | > 16 | | PCAP | 27.1 | |  | |  | |  |  |
| Sweden (56) | 2007-2009 | > 18 | | CAP |  | |  | | 80.8 | |  |  |
| UK (24) | 2008-2013 | > 16 | | CAP | 21.6 | |  | | 56.7^[[23]](#footnote-23)^ | |  |  |
| UK (77) | 2013-2015 | > 16 | | CAP | 6.6 | |  | | 32.5^2^ | | 40.1^[[24]](#footnote-24)^ | 29.9% |
| UK (41) | 2008-2010 | > 16 | | CAP | 19.7 | |  | | 57.4% | |  | 46.4% |
| UK (53) | 2008-2011 | > 16 | | CAP | 27.1 | |  | | 72.5^2^ | |  |  |
| UK (70) | 2008-2013 | > 16 | | PCAP |  | |  | | 57.4 | |  |  |
| UK (79) | 2008-2013 | > 16 | | CAP |  | |  | | 15.2 | |  |  |

**America**

| Country | Period | Age, y | Other information | PCV7 serotypes  (%) | PCV10 serotypes  (%) | PCV13 serotypes  (%) | PPSV23 serotypes  (%) | NVTs  (%) |
| --- | --- | --- | --- | --- | --- | --- | --- | --- |
| Canada(19) | 2003-2011 | > 15 | BP  NBP | 26.6  24 | 11.3^^[[25]](#footnote-25)^^  1^4^ | 65.3  (PCV13 non PCV10^[[26]](#footnote-26)^ = 27.4)  52.9  (PCV13 non PCV10 = 27.9) | 20.9^3^  21.6^3^ | 13.8  25.5 |
| Canada (55) | 2014-2015 | > 16 | PCAP | 0.7 |  | 7.1 | 1.2^3^ | 1.4 |
| Mexico (76) | 2000-2015 | > 18 | Pre PCV7  Post PCV7-PCV13 | 55.5  12.8 |  | 77.8  34.6 | 94.4  60.3 | 0  14.1 |
| US (75) | 2010-2011 | > 50 | CAP | 26.9 |  | 79.5^2^ |  |  |

**Asia**

| Country | Period | Age, y | Other information | PCV7 serotypes  (%) | PCV10 serotypes  (%) | PCV13 serotypes  (%) | PPSV23 serotypes  (%) | NVTs  (%) |
| --- | --- | --- | --- | --- | --- | --- | --- | --- |
| Australia (22) | 1987-2008 | > 14 | BPP |  |  |  | 84.7 |  |
| India (38) | 2013-2015 | >50 | CAP | 16 | 24 | 48 | 66 |  |
| Japan (30) | 2001-2003 | > 16 | CAP |  |  |  | 82.5 |  |
| Japan (31) | 2011-2013  2011  2013 |  | CAP + HCAP + HAP | 46.4  20 |  | 71.4  53.3 | 71.4  60 |  |
| Japan (40) | 2011-2014  2016-2017 | >15 | CAP | 21  10 |  | 53  (PCV13 non PCV7^2^ = 32%)  33  (PCV13 non PCV7 = 23%) | 19 (non PCV13)  18 (non-PCV13) | 28  49 |
| Japan (80) | 2001-2003 | > 20 | CAP |  |  |  | 82.5 |  |
| South Korea (48) | 2015 | > 15 | CAP |  |  | 42.4 | 59.8  (20.7)^2^ |  |

**S7**

**PRISMA 2020 Checklist**

| **Section and Topic** | **Item #** | **Checklist item** | **Location where item is reported** |
| --- | --- | --- | --- |
| **TITLE** | | |  |
| Title | 1 | Identify the report as a systematic review. |  |
| **ABSTRACT** | | |  |
| Abstract | 2 | See the PRISMA 2020 for Abstracts checklist. |  |
| **INTRODUCTION** | | |  |
| Rationale | 3 | Describe the rationale for the review in the context of existing knowledge. |  |
| Objectives | 4 | Provide an explicit statement of the objective(s) or question(s) the review addresses. |  |
| **METHODS** | | |  |
| Eligibility criteria | 5 | Specify the inclusion and exclusion criteria for the review and how studies were grouped for the syntheses. |  |
| Information sources | 6 | Specify all databases, registers, websites, organisations, reference lists and other sources searched or consulted to identify studies. Specify the date when each source was last searched or consulted. |  |
| Search strategy | 7 | Present the full search strategies for all databases, registers and websites, including any filters and limits used. |  |
| Selection process | 8 | Specify the methods used to decide whether a study met the inclusion criteria of the review, including how many reviewers screened each record and each report retrieved, whether they worked independently, and if applicable, details of automation tools used in the process. |  |
| Data collection process | 9 | Specify the methods used to collect data from reports, including how many reviewers collected data from each report, whether they worked independently, any processes for obtaining or confirming data from study investigators, and if applicable, details of automation tools used in the process. |  |
| Data items | 10a | List and define all outcomes for which data were sought. Specify whether all results that were compatible with each outcome domain in each study were sought (e.g. for all measures, time points, analyses), and if not, the methods used to decide which results to collect. |  |
|  | 10b | List and define all other variables for which data were sought (e.g. participant and intervention characteristics, funding sources). Describe any assumptions made about any missing or unclear information. |  |
| Study risk of bias assessment | 11 | Specify the methods used to assess risk of bias in the included studies, including details of the tool(s) used, how many reviewers assessed each study and whether they worked independently, and if applicable, details of automation tools used in the process. |  |
| Effect measures | 12 | Specify for each outcome the effect measure(s) (e.g. risk ratio, mean difference) used in the synthesis or presentation of results. |  |

| Synthesis methods | 13a | Describe the processes used to decide which studies were eligible for each synthesis (e.g. tabulating the study intervention characteristics and comparing against the planned groups for each synthesis (item #5)). |  |
| --- | --- | --- | --- |
|  | 13b | Describe any methods required to prepare the data for presentation or synthesis, such as handling of missing summary statistics, or data conversions. |  |
|  | 13c | Describe any methods used to tabulate or visually display results of individual studies and syntheses. |  |
|  | 13d | Describe any methods used to synthesize results and provide a rationale for the choice(s). If meta-analysis was performed, describe the model(s), method(s) to identify the presence and extent of statistical heterogeneity, and software package(s) used. |  |
|  | 13e | Describe any methods used to explore possible causes of heterogeneity among study results (e.g. subgroup analysis, meta-regression). |  |
|  | 13f | Describe any sensitivity analyses conducted to assess robustness of the synthesized results. |  |
| Reporting bias assessment | 14 | Describe any methods used to assess risk of bias due to missing results in a synthesis (arising from reporting biases). |  |
| Certainty assessment | 15 | Describe any methods used to assess certainty (or confidence) in the body of evidence for an outcome. |  |
| **RESULTS** | | |  |
| Study selection | 16a | Describe the results of the search and selection process, from the number of records identified in the search to the number of studies included in the review, ideally using a flow diagram. |  |
|  | 16b | Cite studies that might appear to meet the inclusion criteria, but which were excluded, and explain why they were excluded. |  |
| Study characteristics | 17 | Cite each included study and present its characteristics. |  |
| Risk of bias in studies | 18 | Present assessments of risk of bias for each included study. |  |
| Results of individual studies | 19 | For all outcomes, present, for each study: (a) summary statistics for each group (where appropriate) and (b) an effect estimate and its precision (e.g. confidence/credible interval), ideally using structured tables or plots. |  |
| Results of syntheses | 20a | For each synthesis, briefly summarise the characteristics and risk of bias among contributing studies. |  |
|  | 20b | Present results of all statistical syntheses conducted. If meta-analysis was done, present for each the summary estimate and its precision (e.g. confidence/credible interval) and measures of statistical heterogeneity. If comparing groups, describe the direction of the effect. |  |
|  | 20c | Present results of all investigations of possible causes of heterogeneity among study results. |  |
|  | 20d | Present results of all sensitivity analyses conducted to assess the robustness of the synthesized results. |  |
| Reporting biases | 21 | Present assessments of risk of bias due to missing results (arising from reporting biases) for each synthesis assessed. |  |
| Certainty of evidence | 22 | Present assessments of certainty (or confidence) in the body of evidence for each outcome assessed. |  |

| **DISCUSSION** | | |  |
| --- | --- | --- | --- |
| Discussion | 23a | Provide a general interpretation of the results in the context of other evidence. |  |
|  | 23b | Discuss any limitations of the evidence included in the review. |  |
|  | 23c | Discuss any limitations of the review processes used. |  |
|  | 23d | Discuss implications of the results for practice, policy, and future research. |  |
| **OTHER INFORMATION** | | |  |
| Registration and protocol | 24a | Provide registration information for the review, including register name and registration number, or state that the review was not registered. |  |
|  | 24b | Indicate where the review protocol can be accessed, or state that a protocol was not prepared. |  |
|  | 24c | Describe and explain any amendments to information provided at registration or in the protocol. |  |
| Support | 25 | Describe sources of financial or non-financial support for the review, and the role of the funders or sponsors in the review. |  |
| Competing interests | 26 | Declare any competing interests of review authors. |  |
| Availability of data, code and other materials | 27 | Report which of the following are publicly available and where they can be found: template data collection forms; data extracted from included studies; data used for all analyses; analytic code; any other materials used in the review. |  |

*From:*  Page MJ, McKenzie JE, Bossuyt PM, Boutron I, Hoffmann TC, Mulrow CD, et al. The PRISMA 2020 statement: an updated guideline for reporting systematic reviews. BMJ 2021;372:n71. doi: 10.1136/bmj.n71. This work is licensed under CC BY 4.0. To view a copy of this license, visit <https://creativecommons.org/licenses/by/4.0/>

1. BPP: Bacteremic Pneumococcal Pneumonia [↑](#footnote-ref-1)
2. NBPP: Non Bacteremic Pneumococcal Pneumonia [↑](#footnote-ref-2)
3. HCAP: Healthcare-associated Pneumonia [↑](#footnote-ref-3)
4. HCAP: Healthcare-associated pneumonia (defined as pneumonia that develops outside the hospital among patients who have had recent substantial exposure to the health care setting) [↑](#footnote-ref-4)
5. HAP: Hospital-acquired pneumonia (defined as pneumonia occurring 48 h or more after admission and not incubating at the time of admission) = represented 5.6% of PP in the study (= 5 patients) [↑](#footnote-ref-5)
6. Vaccinated ≤ 5 years [↑](#footnote-ref-6)
7. Vaccinated ≥ 5 years [↑](#footnote-ref-7)
8. Results distinguish IPP from NIPP serotypes [↑](#footnote-ref-8)
9. Only 3 isolates were isolated from blood [↑](#footnote-ref-9)
10. Serotyping was performed only in invasive strains [↑](#footnote-ref-10)
11. Vaccine is administered only to high-risk children (e.g., children < 2 years) [↑](#footnote-ref-11)
12. PCV10 switched to PCV13 only in Metropolitan Region [↑](#footnote-ref-12)
13. The vaccine was introduced as a pilot vaccination strategy for some of the poorest regions [↑](#footnote-ref-13)
14. Recommended for Indigenous children [↑](#footnote-ref-14)
15. Introduced in the private sector only [↑](#footnote-ref-15)
16. Vaccine is administered only in certain regions of the country [↑](#footnote-ref-16)
17. Vaccine is administered throughout the entire country [↑](#footnote-ref-17)
18. PCV10 was chosen as the refunded vaccine in the NIP, however, it is estimated that a quarter to a third of infants in Poland is vaccinated with PCV13 outside the NIP [↑](#footnote-ref-18)
19. Available in the private market only (has been widely distributed in the pediatric population since 2002) [↑](#footnote-ref-19)
20. PCV13 became the most used vaccine [↑](#footnote-ref-20)
21. PCV vaccination recommendation is regional [↑](#footnote-ref-21)
22. PCAP: Pneumococcal CAP [↑](#footnote-ref-22)
23. % of PP due to 6 additional PCV13 serotypes (1, 3, 5, 6A, 7F, 19A) [↑](#footnote-ref-23)
24. % of PP due to additional PPSV23 serotypes (2, 8, 9N, 10A, 11A, 12F, 15B, 17F, 20, 22F, 33F) [↑](#footnote-ref-24)
25. % of PP due to additional PCV10 (serotypes 1, 5, 7F) [↑](#footnote-ref-25)
26. % of PP due to additional PCV13 nonPCV10 serotypes (3, 6A, 19A) [↑](#footnote-ref-26)
